# The active ingredients: physical activity features linked to healthy brain aging

**DOI:** 10.1186/s13195-026-01998-6

**Published:** 2026-03-05

**Authors:** Claire J. Cadwallader, Pedro Pinheiro-Chagas, Rowan Saloner, Laura Fenton, Anna M. VandeBunte, May Lin, Albert Pham, Coty Chen, Valentina E. Diaz, Molly Olzinski, Sophia Licata, Lana Callies, Carina Lo, Jessica Buxton, Yann Cobigo, Gil Rabinovici, Joel H. Kramer, Kaitlin B. Casaletto, Emily W. Paolillo

**Affiliations:** Memory and Aging Center, Department of Neurology, Weill Institute for Neurosciences, University of California, San Francisco, San Francisco, CA 94158 USA

**Keywords:** Physical activity, Exercise, Step count, Accelerometry, Intensity, Brain aging, Sex differences, Cerebrovascular health, Executive function, White matter

## Abstract

**Background:**

Physical activity is a modifiable lifestyle factor linked to better brain health in older adults, yet the optimal parameters (e.g., frequency, duration), and person-specific interactions (e.g., age, sex), remain unclear.

**Methods:**

We developed a novel algorithm to isolate real-world physical activity “sessions” (at least 10 min at greater than or equal to 40 steps/min) from 30 days of wrist actigraphy data in 279 older adults without dementia. Ridge regression models assessed associations of 42 in- and out-of-session physical activity features and their interactions with demographics on cognition and neuroimaging outcomes.

**Results:**

79% of participants engaged in at least one physical activity session (“exercisers”). Exercisers had lower white matter hyperintensity burden compared to non-exercisers. Session frequency and session step cadence emerged as the most important predictors of brain health, particularly for white matter health indices and executive function, with relatively stronger associations in females. Out-of-session features and all interactions with age were least predictive.

**Conclusions:**

Physical activity session frequency and cadence were the most robust predictors of brain health, emphasizing the importance of physical activity structure over quantity for dementia prevention strategies.

**Supplementary Information:**

The online version contains supplementary material available at 10.1186/s13195-026-01998-6.

## Introduction

Physical activity is widely recognized as a modifiable factor that supports healthy brain aging. Observational studies have linked greater physical activity engagement with reduced dementia risk, better cognitive performance, and preserved brain structure in older adults [1, 2]. However, results from randomized controlled trials (RCTs) of physical activity are mixed [3, 4]. A potential reason for this is our limited understanding of the physical activity features (i.e., intensity, duration) that confer the greatest benefits for brain health. Efforts to understand these optimal features have been constrained by lack of variability in physical activity protocol designs for brain health compounded by challenges with scientific rigor among RCTs (e.g., statistical power, account of baseline performance and/or differences) [5]. Further, inter-individual factors such as age and sex have been shown to moderate physical activity-related brain benefits [6], and are among the top risk factors for dementia [7]. Our current understanding of whether the optimal features of physical activity differ based on these person-specific factors is extremely limited. Addressing knowledge gaps surrounding the importance of specific physical activity features, and for whom, is critical for evidence-based physical activity prescription for dementia prevention.

Identifying important physical activity features for brain health is methodologically challenging. Many prior actigraphy-based studies focused on gross indicators such as total daily step count or time in moderate-to-vigorous intensity [8], which may obscure important nuances such as the structure and frequency of discrete physical activity sessions. Meanwhile, self-report measures suffer from poor precision, recall biases, and limited sensitivity to certain types of activity (e.g., unstructured walking during daily chores) [9]. Although RCTs are the gold standard for causal inference, they are costly and labor-intensive. Further, designing a trial to evaluate the almost innumerable possible combinations of physical activity features is unfeasible. Meta-analyses of RCTs have delivered valuable insights that reinforce the hypothesis that physical activity session parameters influence intervention success. In one study, across intervention modalities (i.e. aerobic, resistance), higher session frequency (≥ 3 sessions/week) and moderate session duration (45–60 min) were associated with the largest benefits for cognitive outcomes in older adults, with the largest effects seen for older adults aged 60–75 years. [10]. Another metanalysis also found that shorter, more frequent sessions may be optimal for cognition in older adults with cognitive impairment; however, this was less clear in a cognitively unimpaired group [11]. Other studies have supported the importance of at least moderate intensity physical activity for cognition and white matter integrity [12–14]. While informative, these studies often categorize parameters that are inherently continuous, resulting in loss of information and reduced sensitivity to detect dose-response relationships. They are also limited by inconsistent reporting of intervention features and subgroup analyses across intervention studies. Further, we are not aware of any studies that have been able to (a) consider between-parameter interactions (e.g., is long session duration only important at low session frequencies?), or (b) assess sex- and age-specific interactions with specific physical activity parameters, and studies that compare intervention parameters for outcomes beyond cognition (e.g., neuroimaging) are scarce.

Advances in wearable technology provide the opportunity to examine rich temporal patterns of real-world movements. Moreover, observational wearable accelerometer studies likely capture habitual, trait-like physical activity patterns that may be more ecologically valid than short-term adherence to a prescribed physical activity protocol [15]. Previous accelerometer studies of older adults have shown that physical activity features representing more frequent transitions between low and high activity, and greater regularity/rhythmicity of activity across days were associated with better cognitive function [16]. Less fragmentation of physical activity (e.g., longer durations and lower probability of transition to sedentary) [17] and vigorous intermittent lifestyle activity (VILPA; brief bouts of vigorous activity) [18] have also been associated with better physical function and reduced mortality. While these findings highlight potentially important parameters, there are limited actigraphy studies attempting to directly characterize real-world engagement in physical activity “sessions” (i.e., discrete episodes of movement) and link them to brain health. Furthermore, some accelerometer-based metrics (e.g., vector magnitudes) can be difficult to interpret, limiting translation into clinically meaningful insights. By contrast, step count is a widely accessible and interpretable metric, already familiar to many individuals through commercial wearable devices [19]. Thus, identifying optimal step-based activity patterns has high translational potential for personalized physical activity recommendations in clinical and public health contexts.

In this study, we developed an objective and scalable method to isolate real-world physical activity “sessions” from minute-level accelerometer-based step count data. We applied this novel approach to a sample of older adults without dementia who wore actigraphy devices for 30 days and completed comprehensive neurobehavioral evaluations, including cognitive assessment and neuroimaging. Outcomes of interest were selected based on those that have previously shown protective associations with physical activity or fitness in older adults including memory [20], executive functioning [21], processing speed [22], medial temporal [23] and frontal [24] lobe volumes, and white matter health indices [25, 26]. The aims were to: (1) identify features of physical activity behavior (e.g., session duration, session frequency, out-of-session activity) most strongly associated with these outcomes, and (2) examine whether age and sex moderate these associations.

## Methods

### Participants

The study included 279 community-dwelling, functionally intact older adults aged 40 and older enrolled in the University of California, San Francisco (UCSF) Memory and Aging Center’s Brain Aging Network for Cognitive Health (BrANCH) study (56% Female, mean age = 72.4 years, SD age = 9.9). All participants were living without dementia based on the clinical dementia rating (CDR) scale (87% CDR = 0, 13% CDR = 0.5) and completed 30 days of wrist-based actigraphy monitoring. Participants were selected based on completion of at least one of the following outcomes within 90 days of their actigraphy monitoring: memory (*n* = 197), executive function (*n* = 266) and/or information processing speed (*n* = 203) testing derived from neuropsychological assessment, and/or brain imaging metrics [medial temporal lobe and frontal lobe volumes (*n* = 182), total white matter hyperintensity burden (*n* = 162), global fractional anisotropy (*n* = 149)]. Exclusion criteria included history or current evidence of the following conditions: diagnosis of DSM-5 major psychiatric disorders, large vessel stroke, multiple sclerosis, epilepsy, significant memory concerns or relative diagnoses, symptomatic neurogenerative disease (e.g., Parkinson’s disease), active substance abuse, HIV, hepatitis C, syphilis, blindness, or deafness. Participants were excluded from completing an MRI scan if they presented with relevant contraindications (e.g., ferromagnetic surgical implants). All participants provided written, informed consent. This study was approved by the UCSF Institutional Review Board and the study protocol was performed in accordance with the ethical standards laid down in the 1964 Declaration of Helsinki and its later amendments.

### Measures

#### Actigraphy monitoring

Participants wore a Fitbit™ wrist actigraphy monitoring device (model: Flex2 or Inspire2; see Table 1) for 30 consecutive days (Fitbit Inc., San Francisco, CA, USA; https://www.fitbit.com). Step count data between these Fitbit models has previously shown strong concordance with each other [27] and with research-grade ActiGraph devices [28, 29]. Continuous minute-level step count data was synced to Fitabase, a research-grade platform for managing wearable device data. To ensure data quality, days where participants recorded fewer than 100 steps were excluded, helping to account for periods of non-wear per previously published methods [30]. All participants had a minimum of 7 days of valid monitoring data, though the average was 28 days. Participants were instructed to engage in their regular physical activity habits during the monitoring period and were blinded to all Fitbit notifications and indication of their daily step count to further limit behavior change.

**Table 1:** Aggregate physical activity features calculated for each participant

Physical activity features	Definition
Session cadence	Average step cadence (steps/min) of a physical activity session across all sessions
Session duration	Average duration (minutes) of a physical activity session across all sessions
Session frequency	Average number of physical activity sessions per week across all weeks
Daily in-session steps	Average number of steps taken within a physical activity session per day across all days
Daily vigorous in-session steps	Average number of steps taken within a physical activity session at a vigorous cadence (≥120 steps/min^8^) per day across all days
Daily out-of-session steps	Average number of steps taken outside of physical activity sessions per day across all days
Daily vigorous out-of-session steps	Average number of steps taken outside of physical activity sessions at a vigorous cadence (≥120 steps/min^8^) per day across all days
Daily moderate-vigorous out-of-session steps (non-exercisers only*)	Average number of steps taken outside of physical activity sessions at a moderate-to-vigorous cadence (≥100 steps/min^8^) per day across all days. *Calculated in non-exercisers due to limited engagement in vigorous steps.

#### Characterization of physical activity sessions and features

**Fig. 1:**
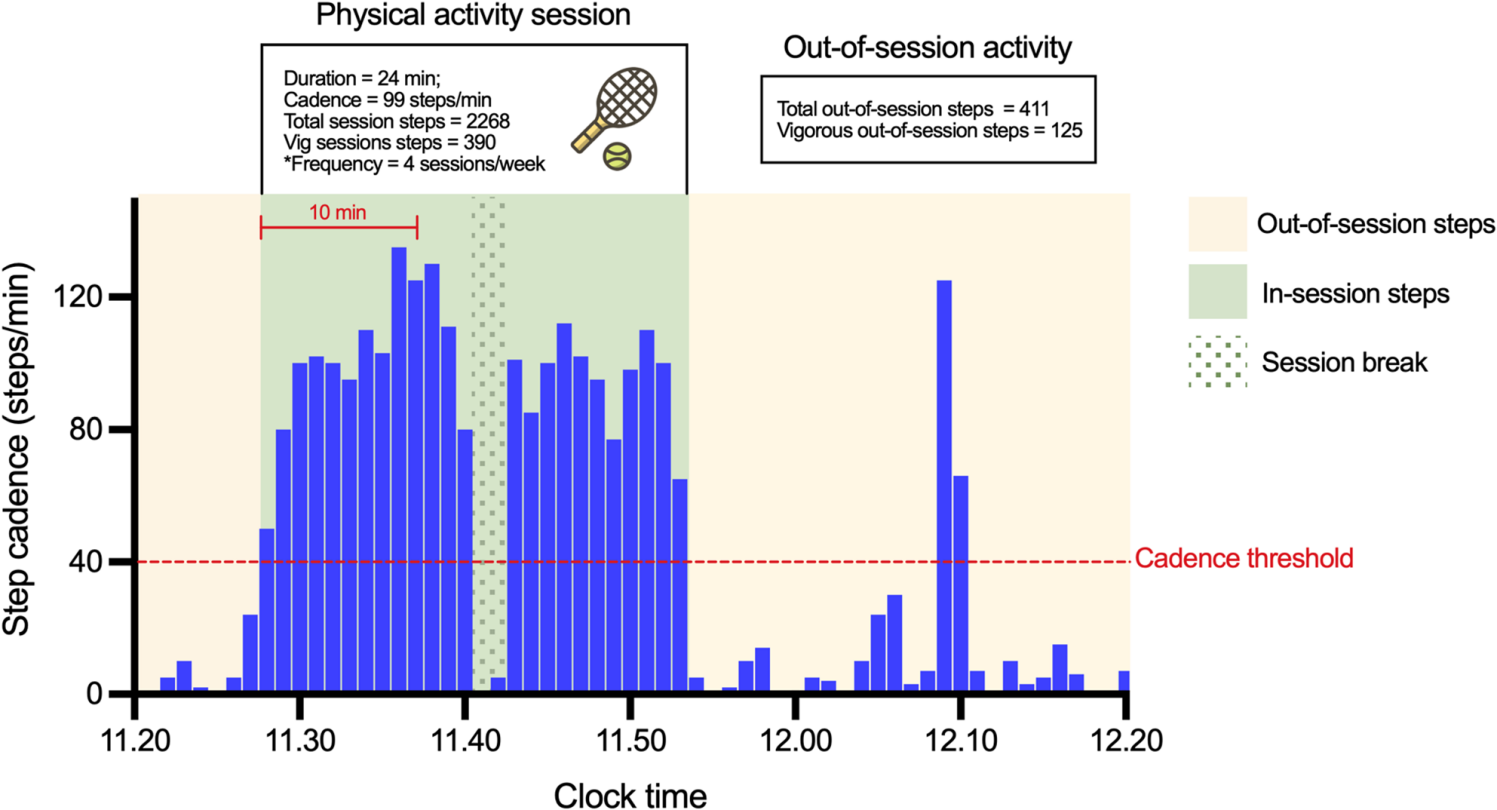
Example of PA session characterization from simulated data for demonstrative purposes. The figure depicts an hour of minute-level step count data from a single individual. A PA session was defined as at least 10 consecutive minutes where step cadence was greater than or equal to 40 steps/min. After the session criteria was met, short breaks (cadence < 40 steps/min) of less than 10 min were allowed within a session. Session features including duration, average cadence, total steps and steps at vigorous cadence were calculated excluding session breaks. *Session frequency is not pictured and was defined as the average number of PA sessions per week. Vig: vigorous (≥120 steps/min^8^)

Physical activity sessions were objectively defined and isolated from the minute-level step count data using a novel algorithm (i.e., a set of rules designed to identify discrete physical activity sessions; Fig. 1). To the best of our knowledge, this is the first study to systematically isolate discrete physical activity sessions from actigraphy data while allowing for realistic interruptions (e.g., brief pauses during activity) that occur during real-world physical activity behavior. Due to lack of current knowledge surrounding the minimum physical activity requirements (e.g., minimum duration, intensity) for brain health outcomes, especially among older adults, we adopted a physical activity session definition that was designed to be as inclusive as possible (Fig. 1). The algorithm defined the beginning of a session by at least 10 consecutive minutes where step cadence was ≥40 steps/min, a threshold considered to represent intentional, low intensity step activity [31]. Once this criterion was met, short “session breaks” (step cadence < 40 steps/min) of less than 10 min were allowed within a session to account for brief interruptions (e.g., resting in between sets of exercises, pausing briefly a walk). A session ended at the last minute where step cadence was ≥40 steps/min and was followed by at least 10 min where step cadence fell below the minimum cadence threshold. To be as inclusive as possible of those who met these criteria, participants were labelled an “exerciser” if they completed at least one physical activity session during the study monitoring period or were labelled a “non-exerciser” if they completed no sessions. We acknowledge that “exercise” is usually defined as engagement in intentional, structured and repetitive physical activity (e.g., going for a recreational walk or run), and that we are unable to confirm that the physical activity sessions we capture are “exercise” versus other forms of physical activity (e.g., incidental steps incurred during activities of daily living) due to the nature of actigraphy data. We have used the label “exerciser” for conciseness. To validate the chosen cadence threshold of ≥40 steps/min, we also iteratively tested different definitions of physical activity sessions following the same process described above but with varying minimum cadence thresholds. More detailed methods and results from this procedure are described in the Supplementary Materials (Table S1).

For each session identified, the following features were characterized: average session cadence (steps/min), session duration (min), total step count, and step count at vigorous cadence (defined as ≥120 steps/min in older adults 8). These session characteristics were calculated excluding breaks that occurred within a session (i.e., time spent in < 40 steps/minute). Out-of-session activity features were also characterized and included: (1) total out-of-session step count/day and (2) out-of-session step count/day at vigorous cadence (≥120 steps/min^8^). For each participant, aggregate features summarizing a person’s overall physical activity patterns across the monitoring period were calculated (Table 1).

#### Neuropsychological evaluation

266 participants completed a comprehensive neuropsychological battery of assessments validated as neuroanatomically sensitive to age-related neurodegenerative processes [32]. Raw scores for each test were transformed to sample-based z-scores. Z-scores were then averaged within cognitive domains to create composite z-scores for episodic memory, executive function and information processing speed, which have been previously published by our group [27, 33].

##### Episodic memory composite

Tests included subtests from the California Verbal Learning Test–Second Edition (CVLT-II) (total immediate recall over all five learning trials, total long delay [20-min] free recall, recognition discriminability) and total delayed [10-min] free recall score of the Benson figure [32]. Episodic memory composite scores for 69 participants were unavailable due to incomplete testing.

##### Executive functioning composite

Tests included the modified Trail Making Test completion time [32], total score from the Stroop interference task (correct items in 60”) [34], a phonemic fluency task (total “D” words generated in 60”) [32], the Delis-Kaplan Executive Function System (D-KEFS) Design Fluency, Condition 1 (total designs generated in 60”), and longest backward digit span (Wechsler Adult Intelligence Scale, Fourth Edition) [35].

##### Information processing speed composite

Processing speed was assessed via computerized visuospatial processing speed tasks programmed on Eprime software (Psychology Software Tools, Inc., Sharpsburg, PA; http://www.pstnet.com/eprime.cfm) that have been described previously [36].

#### Magnetic resonance imaging

##### Acquisition

MRI scans were acquired on a Siemens Trio or Prisma 3 T scanner at UCSF Neuroscience Imaging Center. High-resolution T1-weighted images were collected using MPRAGE sequences (Trio: TR/TE/TI = 2300/2.98/900 ms, Prisma: TR/TE/TI = 2300/2.9/900 ms, α = 9°; 1 mm³ isotropic voxels, sagittal view), followed by visual quality checks to exclude images with motion artifacts. As previously described [37], tissue segmentation used SPM12 [38] and brain volume quantification was performed by transforming a standard parcellation atlas [39] into International Consortium of Brain Mapping (ICBM) space and summing gray matter within parcellated regions of interest. T2/FLAIR images were also collected (Trio: slice thickness = 1.00 mm; slices per slab = 160; in-plane resolution = 0.98 × 0.98 mm; matrix = 256 × 256; TR/TE/TI = 6000/388/2100 ms; flip angle = 120°; Prisma: slice thickness = 1.00 mm; slices per slab = 176; in-plane resolution = 1.0 × 1.0 mm; matrix = 256 × 256; TR/TE/TI = 5000/397/1800 ms; flip angle = 120°), and white matter lesions were segmented by the lesion prediction algorithm [40] from the Lesion Segmentation Tool toolbox (www.statistical-modelling.de/lst.html) for SPM to categorize white matter hyperintensities. Diffusion tensor images (DTI) were collected using a single-shot spin-echo echo-planar imaging (EPI) sequence. DTI data acquired on the Trio 3 T scanner used the following parameters: TR/TE 8200/86 ms; 1 volume B = 0 image and 64 directions at B =2000 s/mm^2^ and an integrated parallel acquisition techniques (iPAT) acceleration factor of 2, 2.2 mm thick slices; 55 slices with in-plane resolution yielding 2.2 mm^3^ isotropic voxels. DTI data acquired on the Prisma 3 T scanner used the following parameters: TR/TE 2420/72 ms; 2 volumes B = 0 image and 10 volumes with 3 multi-shells with 96 directions at B =2500 s/mm^2^; 48 directions at B =1000 s/mm^2^, 30 directions at B =500 s/mm^2^ with an (iPAT acceleration factor of 2 and multi-band acceleration factor of 3), and 2.0 mm thick slices; 69 slices yielding 2.0 mm^3^ isotropic voxels.

##### Primary neuroimaging outcomes

Primary volumetric outcomes of interest in this study included medial temporal lobe (MTL) volume (i.e., bilateral entorhinal, parahippocampal, plus hippocampal volume) and frontal lobe volume. White matter hyperintensity (WMH) volume derived from T2/FLAIR images was used as another primary outcome of interest, representing white matter injury. The primary diffusion metric of interest in this study, global fractional anisotropy (FA), was derived per previously published methods for scans acquired on the Trio and Prisma Scanners separately, then harmonized using the well-validated ComBat method to correct for scanner-specific differences [41].

### Statistical analyses

All data processing, feature engineering, and statistical analyses were conducted using R version 4.4.0. For all parametric analyses performed, outcome variables with a non-normal data distribution were log-transformed. For ridge regression models, outcome variables were pre-adjusted for age and sex (all outcomes), education (cognitive outcomes) and total intracranial volume (sum of gray matter, white matter, and cerebrospinal fluid; neuroimaging outcomes) by extracting residuals (described below in Sect. 2.3.1). For all other analyses, models controlled for these variables via their inclusion as covariates. Two-tailed independent samples t-tests examined sex differences on demographic and clinical characteristics. Wilcoxon signed rank tests with continuity correction were selected to examine differences between in-session and out-of-session step counts within participants due to significant variable distribution skew. ANCOVAs compared exercisers and non-exercisers on brain health outcomes including memory, executive function, processing speed, MTL volume, frontal lobe volume, total WMH burden and global FA. In addition, linear regression analyses with false discovery rate-corrected p values examined bivariate relationships between each physical activity feature and clinical outcomes.

####  Physical activity session feature importance for brain health outcomes in exercisers

To determine the physical activity features most important for brain health, ridge regression machine learning models examined the relative importance of physical activity features (Table 1), their interactions with each other, and their interactions with demographic variables in predicting cognitive and neuroimaging outcomes among exercisers. Importantly, we are not using this analytic approach to develop a predictive model; rather, we utilized ridge regression specifically to understand relative influences of many correlated physical activity features on each brain health outcome. This modeling approach was well-suited to this goal for several reasons. First, many of our physical activity metrics are inherently correlated, and ridge regression effectively addresses multicollinearity by introducing a penalty that shrinks, but does not eliminate, correlated coefficients [42]. This allowed us to retain all candidate features in the model, preserving potentially meaningful multicollinear predictors that might otherwise be randomly excluded by variable selection methods such as LASSO or elastic net. Additionally, ridge regression offers better interpretability of interaction terms while mitigating overfitting compared to other machine learning algorithms like random forest, especially in high-dimensional data sets [42].

Ridge regression models were implemented using the glmnet package in R. Predictors included all aggregate physical activity features (Table 1), and their pairwise interaction terms with each other, age, and sex (42 predictor terms in total). Predictor and outcome variables were standardized prior to model fitting, allowing resulting ridge regression coefficient magnitudes to be compared directly as indicators of relative importance on each outcome. To focus on the unique predictive importance of physical activity features for brain health outcomes over and above the influence of demographic variables, which are known to associate strongly with cognition and neuroimaging measures, outcomes were pre-adjusted for age and sex (all outcomes), education (cognitive outcomes), and total intracranial volume (neuroimaging outcomes) by regressing each outcome on these covariates and extracting the residuals. These residualized outcomes were used in the subsequent ridge regression models. We performed 10-fold cross-validation using the ‘caret’ package [43] in R to identify the optimal penalty parameter (lambda) based on the minimum mean cross-validated error. Final models were trained using the optimal lambda, and standardized coefficients were extracted for interpretation. Model performance was evaluated using R-squared and mean absolute error (MAE). To estimate the stability of model performance, we conducted non-parametric bootstrapping with 1000 resamples to derive percentile-based confidence intervals around R-squared values. In addition, to quantify the relative importance of different physical activity features in the models, we summed the absolute standardized coefficients within each model and calculated the proportion attributable to each predictor term. We also calculated the relative contribution of in-session features (the proportion of the total sum of coefficient weights attributable to all in-session terms excluding interaction terms with out-of-session features) and out-of-session features (the proportion of the total sum of coefficient weights attributable to out-of-session terms excluding interaction terms with out-of-session features) within each model. Finally, sensitivity analyses included a) running each ridge regression model with only the seven core physical activity features (listed in Table 1) and no interaction terms to examine the robustness of physical activity feature rankings for each outcome, and b), adding number of valid Fitbit wear days to each ridge regression model as a covariate.

####  Physical activity in non-exercisers

Among non-exercisers, linear regression models were used to assess the relationship between average total daily step count (i.e. out-of-session step count) and average daily step count at moderate-to-vigorous cadence (i.e. out-of-session step count at moderate-to-vigorous cadence) with cognitive and neuroimaging outcomes. Models covaried for age and sex (all outcomes), education (cognitive outcomes) and total intracranial volume (neuroimaging outcomes).

## Results

**Fig. 2:**
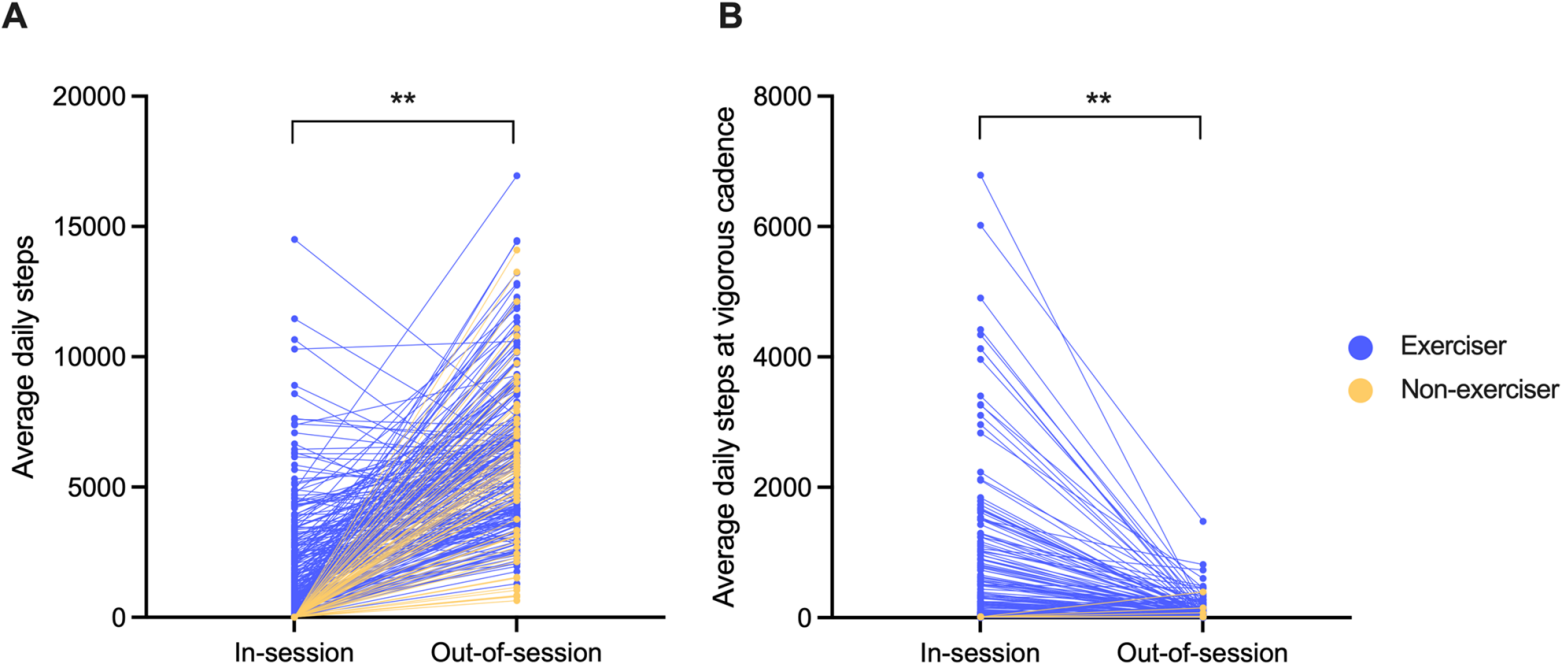
Comparison of in-session vs. out-of-session step counts for each participant (represented by unique lines) for (**A**) average daily total steps and (**B**) average daily steps at vigorous cadence. Exercisers were defined as participants who engaged in at least one physical activity session during the study monitoring period. Significant differences between in-session and out-of-session step counts remained consistent when non-exercisers were excluded. Wilcoxon signed rank tests with continuity correction were used to examine step count differences due to variable distribution skew. **p = < 0.001

Descriptive characteristics of the sample are presented in Table 2. Of the total sample, 79% engaged in at least one physical activity session during the monitoring period (“exercisers”), participating in an average of four physical activity sessions per week. On average, males had greater years of education and faster information processing speed scores compared to females. In contrast, females had higher memory scores and higher session cadence compared to males. On average, participants engaged in more out-of-session steps (78% of total step count) than in-session steps (ηp^2^ *=* 0.61, p = < 0.001, Fig. 2A), even when excluding non-exercisers (ηp^2^ *=* 0.57, p = < 0.001). In addition, participants engaged in more steps at vigorous cadence in-session compared to out-of-session (ηp^2^ *=* 0.15, p = < 0.001, Fig. 2B), even when excluding non-exercisers (ηp^2^ *=* 0.20, p = < 0.001). ANCOVAs covarying for age, sex, education and total intracranial volume showed that participants who engaged in physical activity sessions had lower WMH burden compared to non-exercisers (ηp^2^ *=* 0.13, *p* = 0.005) but did not differ on cognitive or other imaging outcomes (all *p* > 0.121). Results from bivariate analyses between each physical activity feature and brain health outcomes are reported in the supplementary materials (Table S2); these analyses broadly showed that session cadence, duration, and frequency all tended to have the strongest bivariate associations with executive functioning and white matter health.

**Table 2:** Study sample demographic and clinical characteristics

	Full sample	Male	Female	Independent Samples T-Test
Demographic/clinical features				
Total n	279	44% (122)	56% (157)	-
Age (years)	72.44 (9.93)	72.98 (10.17)	72.03 (9.75)	t(277) = 0.79, *p* = 0.432
Global CDR, % score of 0.5	13% (36)	18% (22)	9% (14)	-
Education (years)	17.50 (2.24)	17.87 (2.26)	17.19 (2.18)	**t(277) = 2.49**,*p* = 0.013
Race				
White	81% (226)	84% (103)	78% (123)	-
Black	< 2% (5)	< 1% (1)	3% (4)	-
Asian	12% (33)	11% (13)	13% (20)	-
Other	6% (15)	4% (5)	6% (10)	-
Memory (z-score; *n* = 197)	−0.07 (0.82)	−0.22 (0.90)	0.05 (0.74)	**t(195)=−2.35**, *p* = 0.020
Executive function (z-score; *n* = 266)	0.05 (0.72)	0.04 (0.72)	0.06 (0.72)	t(264)=−0.16, *p* = 0.876
Processing speed (z-score; *n* = 203)	2.45 (1.48)	2.74 (1.48)	2.23 (1.45)	**t(201) = 2.14**,*p* = 0.034
Medial temporal lobe volume (mm^3^, *n* = 182)	9664 (1028)	10,201 (1030)	9271 (835)	*t(180) = 1.41, *p* = 0.159
Frontal lobe volume (mm^3^, *n* = 182)	65,118 (7006)	68,755 (6653)	62,451 (6009)	*t(180) = 0.26, *p* = 0.796
Total white matter hyperintensity burden (mm^3^, *n* = 162)	3620 (4926)	3523 (4997)	3688 (4901)	*t(160)=−1.81, *p* = 0.073
Global fractional anisotropy (*n* = 149)	0.44 (0.03)	0.43 (0.03)	0.44 (0.02)	*t(147)=−0.65, *p* = 0.518
Physical activity features				
Valid days of step count data	28 (9)	27 (10)	29 (8.8)	t(277)=−1.24, *p* = 0.218
Fitbit model, % Inspire2	55% (154)	59% (72)	52% (82)	-
Exercisers, %	79% (220)	81% (99)	77% (121)	-
Daily out-of-session steps	6057 (2874)	6375 (2809)	5811 (2909)	t(277) = 1.63, *p* = 0.104
Daily in-session steps	1703 (2234)	1691 (2045)	1713 (2378)	t(277)=−0.08, *p* = 0.936
Daily vigorous out-of-session steps	56 (139)	46 (148)	63 (131)	t(277)=−1.00, *p* = 0.319
Daily vigorous in-session steps	452 (993)	407 (948)	487 (1028)	t(277)=−0.66, *p* = 0.507
Session duration (mins)**	25 (18)	26 (17)	24 (18)	t(218) = 1.18, *p* = 0.241
Session cadence (steps/min)**	90 (11)	88 (11)	92 (12)	**t(218)=−2.33**,*p* = 0.021
Session frequency (sessions/week)	4 (4)	4 (4)	4 (5)	t(277) = 0.32, *p* = 0.098

Mean (SD) or % (n) reported

*T-tests to assess sex differences in neuroimaging variables adjusted for total intracranial volume

**Session duration and cadence variables were calculated for exercisers only

Independent samples t-tests compared males and females, and statistically significant tests are represented in bold (*p*<0.05)

### Physical activity session feature importance for brain health outcomes among exercisers

**Fig. 3:**
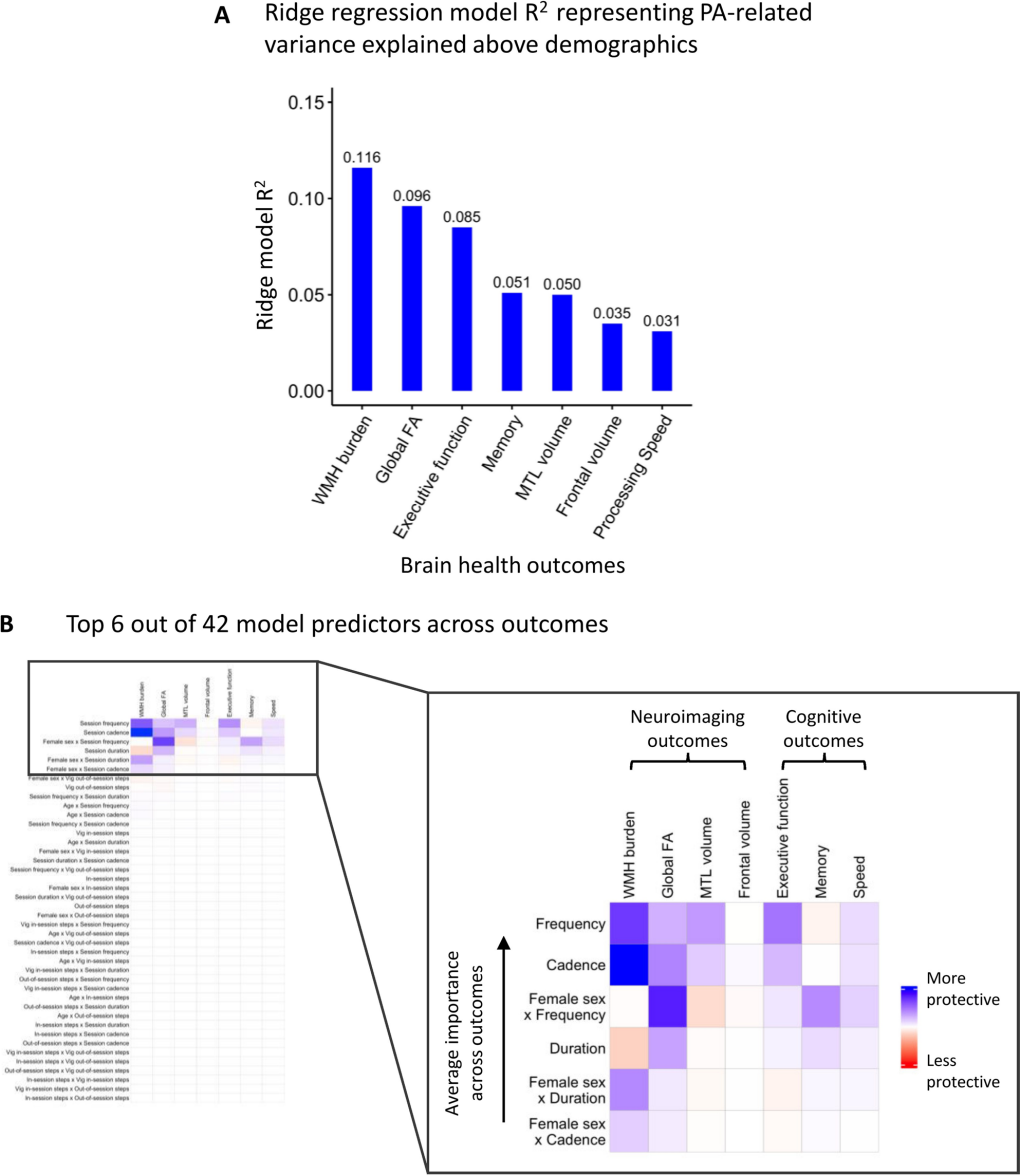
Ridge regression models with λ optimized via 10-fold cross-validation examined the relative importance of physical activity features on brain health outcomes among exercisers. **A** Ridge model R^2^ values representing the added variance explained beyond demographic factors and intracranial volume (for neuroimaging outcomes) are presented for each outcome. **B** A heatmap of standardized model coefficients for the top six predictors on average across outcomes. While the absolute value of the coefficients in ridge regression models are not interpretable as unbiased effect sizes due to regularization, the magnitude of these coefficients is interpretable as relative association strengths compared to other predictors in the model. Rows of the heatmap are ordered to reflect the average importance ranking of physical activity session features across outcomes in descending order (top to bottom). Interaction terms represent the moderating effect of female sex compared to male sex on the association between physical activity session features and the outcome. *WMH* White matter hyperintensity, *FA* Fractional anisotropy

In exercisers, ridge regression models were used to identify specific physical activity features with the greatest predictive importance for cognition and imaging outcomes. Figure 3 displays: (A) the model R^2^ values representing the variance in brain health outcomes explained by physical activity features beyond the influence of demographic variables, and (B) the topmost important predictors across outcomes. A full heatmap including all 42 predictor terms is available in the supplementary materials (Fig. S1). Across models, Session Frequency and Session Cadence consistently emerged among the most important predictors, showing protective associations with brain health outcomes, while out-of-session features were consistently among the least important predictors. For example, out-of-session features accounted for an average of only 3% (range 2–5%) of the total coefficient weights across models. The model for WMH burden achieved the highest R^2^ of 0.116 (95%CI: 0.045, 0.500; MAE: 0.638), meaning physical activity features cumulatively explained 11.6% of variance in WMH volume, with Session Cadence and Session Frequency emerging as the top predictors (accounting for 38% and 27% of total coefficient weights, respectively). Next, physical activity features explained 9.6% of variance in global FA (R^2^ = 0.096; 95%CI: 0.055, 0.509; MAE: 0.660), with top predictors including the interaction between Sex x Session Frequency (a stronger protective association was observed in females compared to males) and Session Cadence (accounting for 40% and 21% of total coefficient weights, respectively). Physical activity features explained 8.5% of variance in executive function (R^2^ = 0.085; 95%CI: 0.053, 0.326; MAE: 0.516), again with Session Frequency and Session Cadence emerging as the top predictors (accounting for 50% and 24% of total coefficient weights, respectively). Physical activity features explained about 5% or less variance in the remaining outcomes, including memory (R^2^ = 0.051; 95%CI: 0.038, 0.437; MAE: 0.609), medial temporal lobe volume (R^2^ = 0.050; 95%CI: 0.041, 0.389; MAE: 0.767), frontal lobe volume (R^2^ = 0.035; 95%CI: 0.028, 0.368; MAE: 0.798), and processing speed (R2 = 0.031; 95%CI: 0.017, 0.334; MAE: 0.772); nonetheless, these models converged with the top predictors from our other brain health outcomes including Session Cadence, Session Frequency, and the interaction between Sex x Session Frequency. Sensitivity analyses examining the robustness of physical activity feature rankings when removing all interaction terms from the models showed highly consistent feature rankings, including session frequency and cadence as the top predictors on average across outcomes and out-of-session features among the least predictive features (Table S3). In addition, when including number of valid Fitbit wear days as a covariate in the models, rank ordering of features also remained consistent.

### Physical activity in non-exercisers

Although non-exercisers did not engage in sustained bouts of movement above a 40-step cadence threshold for at least 10 min, they still averaged 6124 total steps/day (SD = 3190) and 53 moderate-to-vigorous steps per day (SD = 229). Thus, we examined associations between these physical activity features and brain health outcomes in non-exercisers, covarying for age and sex (all outcomes), education (cognitive outcomes) and total intracranial volume (neuroimaging outcomes; Table 3). Higher average daily total step count showed protective associations with medial temporal lobe volume (β = 0.48, *p* = 0.004), WMH burden (β = −0.53, *p* = 0.003) and global FA (β = 0.42, *p* = 0.016). Average daily moderate-to-vigorous step count was positively associated with memory (β = 0.39, *p* = 0.006). Together, these results suggest that greater physical activity, even when not sustained for at least 10 min at a time, may still be beneficial for brain health.

**Table 3:** Linear regression models examining physical activity features in non-exercisers

	Daily total steps		Daily MV steps	
	β (SE)	*p*	β (SE)	*p*
Memory (*n* = 47)	0.19 (0.10)	0.073	1.08 (0.38)	**0.006**
Executive function (*n* = 54)	0.13 (0.09)	0.142	−0.22 (0.27)	0.421
Processing speed (*n* = 47)	0.07 (0.14)	0.643	0.09 (0.12)	0.446
MTL volume (*n* = 35)	0.46 (0.15)	**0.004**	−0.36 (0.48)	0.451
Frontal lobe volume (*n* = 35)	0.11 (0.17)	0.526	−0.46 (0.53)	0.397
Total WMH burden* (*n* = 32)	−0.51 (0.15)	**0.003**	−0.36 (0.57)	0.530
Global FA (*n* = 33)	0.39 (0.15)	**0.016**	−0.32 (0.57)	0.583

Linear regression models each examined the association of average daily total step count (i.e. daily out-of-session step count) and average daily moderate-vigorous (MV) step count (i.e. daily out-of-session step count at MV cadence) with brain health outcomes. Outcomes were pre-adjusted for covariates including age and sex (all outcomes), education (cognition outcomes) and total intracranial volume (imaging outcomes)

*Total WMH burden values were log transformed due to distribution skew

*MTL* Medial temporal lobe, *FA* Fractional anisotropy

Bolded p values represent statistical significance of p < 0.05

## Discussion

The way physical activity is structured, rather than simply how much is accumulated, may be a key determinant of its benefits for the aging brain. Our study is the first to apply an objective and scalable algorithm to identify and characterize real-world physical activity “sessions” from objective wearable step count data and to link these to multimodal markers of brain health in older adults, with consideration of potential interactions with age and sex. We showed that engagement in physical activity sessions of at least 10 min and at least low intensity (≥40 steps/min) was associated with lower white matter hyperintensity burden, and that higher session frequency and step cadence consistently showed the most protective relationships with brain health outcomes, particularly for white matter health indicators (white matter hyperintensity burden, global fractional anisotropy) and executive function. These associations were relatively stronger for females compared to males. Although engagement in physical activity sessions was associated with better brain health, there were still positive associations between total activity metrics with brain health outcomes in those who did not engage in any sessions, supporting the concept that any activity is better than no activity for brain health.

A key finding of the study was that while most participants engaged in most of their daily steps outside of physical activity sessions, out-of-session features were consistently among the least important predictors of brain health outcomes in exercisers. This finding suggests that the presence and structure of in-session activity may be the most potent driver of brain health in these individuals, nuance that is lost when traditional aggregate measures of physical activity are used (e.g., total daily step count). It is important to note that the relative importance of in-session versus out-of-session features may differ as a function of overall physical activity level. Among non-exercisers who did not engage in any physical activity sessions, any accumulated physical activity showed beneficial associations with brain health outcomes such as MTL volume and white matter health, supporting the principle that any activity is better than no activity for brain health. This interpretation is consistent with physical activity dose-response research showing that the greatest health benefits occur when transitioning from inactive to minimally active, with diminishing returns at higher activity levels [44, 45]. Our findings suggest that for individuals who do not engage in any physical activity sessions, any increase in daily steps shows meaningful associations with brain health, whereas among those with physical activity session engagement, optimizing specific session characteristics may be most influential for maximizing brain health benefits.

In this study, the most important physical activity features showed the largest associations with outcomes often linked to cerebrovascular health [46, 47]. Specifically, engagement in any physical activity sessions and higher session frequency and cadence were most predictive of better white matter health indicators and executive function. There is an abundance of evidence supporting potential mechanisms of structured physical activity sessions for cerebrovascular-related outcomes. Regular engagement in physical activity is shown to promote angiogenesis [48], improve endothelial cell function [49] and cerebral perfusion [50], and reduce arterial stiffness [51], inflammation [52] and metabolic risk factors [53]. These effects reduce ischemic stress on white matter in the brain, preserving myelin integrity [54]. Furthermore, physical activity-related enhancement of neurovascular coupling may support metabolic needs during cognitive tasks [55], particularly executive control networks that show age-related vulnerability [56]. In sum, results suggest cerebrovascular-related pathways may be key targets of physical activity features, including those we found most important such as higher session frequency and intensity, to support brain health.

To our knowledge, we are the first study to examine potential interactions between specific physical activity session parameters and sex on a broad range of brain health outcomes. While session features showed protective associations with brain health outcomes regardless of sex, these relationships were relatively stronger in females compared to males for some outcomes, including indicators of white matter health. This is consistent with a growing body of evidence that suggests there may be sex-specific effects of physical activity for brain heath. In females, physical activity has been associated with greater cortical perfusion [57], better executive function [58, 59], and larger increases in dorsolateral prefrontal cortex volumes [59] compared to males. In contrast, studies examining hippocampal volumes have been mixed [59, 60]. Some have also found male-specific associations of physical activity, including lower amyloid burden [57] and lower plasma glial fibrillary acidic protein, a marker of neuroinflammation [27]. Potential reasons for these sex-dependent benefits include differences in the effects of physical activity on sex hormone levels seen between females and males [6], and direct interactions between sex hormones and neurotrophic factors such as BDNF that are hypothesized to underly the benefits of physical activity [61]. In addition, females have substantially lower risk of cardiovascular disease than males until the menopausal transition, after which risk rises significantly, precipitated by dramatic changes in sex hormones like estrogen [62, 63]. It is possible that the greater protective associations between session features and cerebrovascular-related outcomes in older females is related to this increased cardiovascular risk profile.

It is also pertinent to consider other interactions that showed minimal importance for brain health outcomes in this study. Pairwise interactions between different PA features (e.g., between session frequency and session cadence) were consistently among the least important model terms. This suggests that session features function more independently for brain health; or in other words, frequency of participation does not make up for intensity of participation, and vice versa. For example, engaging in one or two very high intensity sessions or sessions of long duration may not compensate for the detrimental effects of having overall low session frequency, and/or that engaging in highly frequent, high intensity sessions does not necessarily confer synergistically larger benefits. Interactions between physical activity parameters on brain health outcomes have not been previously explored in depth. Here we provide evidence that these may be less important considerations when developing prescription recommendations for older adults. Interactions between physical activity features and age were also consistently among the least important model terms. This suggests that the importance of features like increasing session frequency and cadence may be relevant for brain health across the age spectrum represented in our sample (40–91 years). Previous studies examining age as a moderator of the benefits of engagement in any physical activity for cognition have resulted in conflicting findings. Some suggest attenuated benefits in the oldest age categories (i.e., 70–75 + years) [64, 65], while another found the strongest benefits of physical activity interventions in “middle-old” samples (60–75 years) [10], and others have shown no moderating effect of age on physical activity intervention outcomes [66] or cognitive trajectories [67]. However, there is a scarcity of studies that have included brain health outcomes beyond cognition, and to date, none have examined interactions of age with specific physical activity parameters (e.g., session frequency, duration). Our results suggest that features including higher session frequency and cadence are associated with a range of brain health outcomes in older adults regardless of increasing age. This may help to simplify public health messaging around person-specific physical activity recommendations.

There are several important strengths and limitations of the study design. Firstly, measuring physical activity via wrist worn actigraphy with a minute-level step count sampling frequency may be less accurate at capturing the true intensity of some types of physical activity behaviors, e.g., cycling, resistance exercises. Therefore, while cadence metrics are regularly used as a proxy for intensity [8], our step cadence features (e.g., session cadence) may under-represent the true intensity of physical activity for some participants who regularly engaged in activities other than walking/running. The use of step-count actigraphy data also meant that we were unable to determine the “mode” of physical activity participants were engaging in (e.g., aerobic, resistance), which will be important to examine in future studies with the addition of activity logs. While our conclusions are limited to step count activity, step count is a highly interpretable metric that is accessible to many individuals via popular commercial wearable devices, boosting the translational potential of step count-based recommendations. Second, the physical activity data in this study were observational, limiting conclusions related to directionality and causality. However, these data likely reflect habitual physical activity patterns and capture physical activity bouts during activities of daily living (e.g., walking to the bus station) that are often unaccounted for in RCT trials. Furthermore, this approach allowed us to model naturalistic physical activity session features continuously, increasing sensitivity to detect dose-dependent relationships. Third, session frequency and cadence consistently showed higher relative contributions to model predictions compared to other physical activity features, however, the moderate model R² values (ranging from 3% to 12%) and wide confidence intervals indicate that these features explain meaningful but modest variance in brain health outcomes. Fourth, while our results identify session frequency and cadence as important features for brain health, we are unable to provide guidance on specific targets that are associated with clinically meaningful outcomes (i.e., how frequent is frequent enough?). Our study provides the opportunity to observe patterns of physical activity behavior that are most strongly associated with brain health outcomes, and that can be prioritized for further investigation of specific targets in future RCTs. In the interim, our findings could be used in combination with results from meta-analyses reporting a “minimum total dose” of physical activity associated with clinically meaningful improvements in cognition [68], prioritizing the prescription of shorter sessions at higher frequency and intensity to achieve this minimum target.

## Conclusions

In conclusion, any physical activity may be better than no activity but engagement in some physical activity sessions of at least 10 min and at least low intensity is associated with lower white matter injury in older adults without dementia. Further, regardless of increasing age, engaging in shorter sessions of at least 10 min but at higher frequency and intensity may be optimal, particularly for outcomes related to white matter health and executive function.

## Supplementary Information

Supplementary Material 1

## Data Availability

Deidentified data supporting the conclusions of this article will be made available on request from any qualified investigator (https://memory.ucsf.edu/research-trials/professional/open-science#Data-Sharing). UCSF Memory and Aging Center data requests can be sent to the corresponding author.

## Abbreviations

RCTs: Randomized controlled trials
VILPA: Vigorous intermittent lifestyle physical activity
UCSF: University of California, San Francisco
BrANCH: Brain Aging Network for Cognitive Health
CDR: Clinical dementia rating
CVLT-II: California Verbal Learning Test–Second Edition
D-KEFS: Delis-Kaplan Executive Function System
MRI: Magnetic resonance imaging
ICBM: International Consortium of Brain Mapping
DTI: Diffusion tensor imaging
EPI: Echo-planar imaging
iPAT: Integrated parallel acquisition techniques
MTL: Medial temporal lobe
TIV: Total intracranial volume
WMH: White matter hyperintensity
FA: Fractional anisotropy
MAE: Mean absolute error

## References

[1] Blondell SJ, Hammersley-Mather R, Veerman JL. Does physical activity prevent cognitive decline and dementia? A systematic review and meta-analysis of longitudinal studies. BMC Public Health. 2014;14(1):1–12. 24885250 10.1186/1471-2458-14-510 PMC4064273

[2] Erickson KI, Voss MW, Prakash RS, et al. Exercise training increases size of hippocampus and improves memory. Proc Natl Acad Sci USA. 2011;108(7):3017–22. 21282661 10.1073/pnas.1015950108 PMC3041121

[3] Young J, Angevaren M, Rusted J, Tabet N. Aerobic exercise to improve cognitive function in older people without known cognitive impairment. *Cochrane Database of Systematic Reviews*. 2015;(4):CD005381. 10.1002/14651858.CD005381.pub4 PMC10554155 25900537

[4] Zhang W, Zhou C, Chen A. A systematic review and meta-analysis of the effects of physical exercise on white matter integrity and cognitive function in older adults. Geroscience. 2024;46(2):2641–51. 38108993 10.1007/s11357-023-01033-8 PMC10828294

[5] Ciria LF, Román-Caballero R, Vadillo MA, et al. An umbrella review of randomized control trials on the effects of physical exercise on cognition. Nat Hum Behav. 2023;7(6):928–41. 36973359 10.1038/s41562-023-01554-4

[6] Barha CK, Hsu CL, ten Brinke L, Liu-Ambrose T. Biological sex: a potential moderator of physical activity efficacy on brain health. Front Aging Neurosci. 2019;11:498762. 10.3389/fnagi.2019.00329 PMC6908464 31866852

[7] Riedel BC, Thompson PM, Brinton RD. Age, APOE and sex: triad of risk of Alzheimer’s disease. J Steroid Biochem Mol Biol. 2016;160:134–47. 26969397 10.1016/j.jsbmb.2016.03.012 PMC4905558

[8] Tudor-Locke C, Mora-Gonzalez J, Ducharme SW, et al. Walking cadence (steps/min) and intensity in 61–85-year-old adults: the CADENCE-Adults study. Int J Behav Nutr Phys Activity. 2021;18(1):1–12. 10.1186/s12966-021-01199-4 PMC8461976 34556146

[9] Prince SA, Adamo KB, Hamel ME, Hardt J, Gorber SC, Tremblay M. A comparison of direct versus self-report measures for assessing physical activity in adults: a systematic review. Int J Behav Nutr Phys Act. 2008;5(1):1–24. 18990237 10.1186/1479-5868-5-56 PMC2588639

[10] Han H, Zhang J, Zhang F, Li F, Wu Z. Optimal exercise interventions for enhancing cognitive function in older adults: a network meta-analysis. Front Aging Neurosci. 2025;17:1510773. 40717897 10.3389/fnagi.2025.1510773 PMC12289702

[11] Sanders LMJ, Hortobágyi T, van Gemert S la B, Van Der Zee EA, Van Heuvelen MJG. Dose-response relationship between exercise and cognitive function in older adults with and without cognitive impairment: a systematic review and meta-analysis. PLoS One. 2019;14(1):e0210036. 10.1371/journal.pone.0210036 PMC6328108 30629631

[12] Xu L, Gu H, Cai X, et al. The effects of exercise for cognitive function in older adults: a systematic review and meta-analysis of randomized controlled trials. Int J Environ Res Public Health. 2023;20(2):1088. 36673844 10.3390/ijerph20021088 PMC9858649

[13] Northey JM, Cherbuin N, Pumpa KL, Smee DJ, Rattray B. Exercise interventions for cognitive function in adults older than 50: a systematic review with meta-analysis. Br J Sports Med. 2018;52(3):154–60. 28438770 10.1136/bjsports-2016-096587

[14] Pani J, Eikenes L, Reitlo LS, Stensvold D, Wisløff U, Håberg AK. Effects of a 5-year exercise intervention on white matter microstructural organization in older adults. A generation 100 substudy. Front Aging Neurosci. 2022;14:859383. 35847676 10.3389/fnagi.2022.859383 PMC9278017

[15] Wright SP, Hall Brown TS, Collier SR, Sandberg K. How consumer physical activity monitors could transform human physiology research. Am J Physiol Regul Integr Comp Physiol. 2017;312(3):R358–67. 28052867 10.1152/ajpregu.00349.2016 PMC5401997

[16] Fan LJ, Wang FY, Zhao JH, et al. From physical activity patterns to cognitive status: development and validation of novel digital biomarkers for cognitive assessment in older adults. Int J Behav Nutr Phys Activity. 2025;22(1):1–15. 10.1186/s12966-025-01706-x PMC11748278 39833903

[17] Wilson JJ, McMullan I, Blackburn NE, et al. The Association of Physical Activity Fragmentation with Physical Function in Older Adults: Analysis from the SITLESS Study. J Ageing Longev. 2022;2(1):63–73.

[18] Stamatakis E, Ahmadi MN, Gill JMR, et al. Association of wearable device-measured vigorous intermittent lifestyle physical activity with mortality. Nat Med. 2022;28(12):2521–9. 36482104 10.1038/s41591-022-02100-x PMC9800274

[19] Bassett DR, Toth LP, LaMunion SR, Crouter SE. Step counting: a review of measurement considerations and health-related applications. Sports Med. 2017;47(7):1303–15. 28005190 10.1007/s40279-016-0663-1 PMC5488109

[20] Aghjayan SL, Bournias T, Kang C, et al. Aerobic exercise improves episodic memory in late adulthood: a systematic review and meta-analysis. Commun Med. 2022;2(1):15. 35603310 10.1038/s43856-022-00079-7 PMC9053291

[21] Colcombe S, Kramer AF. Fitness effects on the cognitive function of older adults: a meta-analytic study. Psychol Sci. 2003;14(2):125–30. 12661673 10.1111/1467-9280.t01-1-01430

[22] Colcombe S, Kramer AF. Fitness effects on the cognitive function of older adults: a meta-analytic study. Psychol Sci. 2003;14(2):125–130. 12661673 10.1111/1467-9280.t01-1-01430

[23] Erickson KI, Prakash RS, Voss MW, et al. Aerobic fitness is associated with hippocampal volume in elderly humans. Hippocampus. 2009;19(10):1030–9. 19123237 10.1002/hipo.20547 PMC3072565

[24] Northey JM, Rattray B, Pumpa KL, et al. Objectively measured physical activity is associated with dorsolateral prefrontal cortex volume in older adults. Neuroimage. 2020;221:117150. 32668298 10.1016/j.neuroimage.2020.117150

[25] Zhang W, Zhou C, Chen A. A systematic review and meta-analysis of the effects of physical exercise on white matter integrity and cognitive function in older adults. Geroscience. 2023;46(2):2641–51. 38108993 10.1007/s11357-023-01033-8 PMC10828294

[26] Tseng BY, Gundapuneedi T, Khan MA, et al. White matter integrity in physically fit older adults. NeuroImage. 2013;82:510–6. 23769914 10.1016/j.neuroimage.2013.06.011 PMC3759589

[27] Cadwallader CJ, VandeBunte AM, Fischer DL, et al. BDNF Val66Met polymorphism moderates associations between physical activity and neurocognitive outcomes in older adults. Alzheimer’s Dementia: Translational Res Clin Interventions. 2025;11(2):e70106. 10.1002/trc2.70106 PMC12178939 40547329

[28] Delobelle J, Lebuf E, Dyck DV, et al. Fitbit’s accuracy to measure short bouts of stepping and sedentary behaviour: validation, sensitivity and specificity study. DIGITAL HEALTH. 2024. 10.1177/20552076241262710. 38894943 10.1177/20552076241262710 PMC11185038

[29] Chu AHY, Ng SHX, Paknezhad M, et al. Comparison of wrist-worn Fitbit Flex and waist-worn ActiGraph for measuring steps in free-living adults. PLoS One. 2017;12(2):e0172535. 28234953 10.1371/journal.pone.0172535 PMC5325470

[30] Paolillo EW, Lee SY, VandeBunte A, et al. Wearable Use in an Observational Study Among Older Adults: Adherence, Feasibility, and Effects of Clinicodemographic Factors. Front Digit Health. 2022;4:884208. 35754462 10.3389/fdgth.2022.884208 PMC9231611

[31] Tudor-Locke C, Camhi SM, Leonardi C, et al. Patterns of adult stepping cadence in the 2005–2006 NHANES. Prev Med (Baltim). 2011;53(3):178–81. 10.1016/j.ypmed.2011.06.004 21708187

[32] Kramer JH, Jurik J, Sharon JS, et al. Distinctive neuropsychological patterns in frontotemporal dementia, semantic dementia, and Alzheimer disease. Cogn Behav Neurol. 2003;16(4):211–8. 14665820 10.1097/00146965-200312000-00002

[33] Staffaroni AM, Brown JA, Casaletto KB, et al. The longitudinal trajectory of default mode network connectivity in healthy older adults varies as a function of age and is associated with changes in episodic memory and processing speed. J Neurosci. 2018;38(11):2809–17. 29440553 10.1523/JNEUROSCI.3067-17.2018 PMC5852659

[34] Stroop JR. Studies of interference in serial verbal reactions. J Exp Psychol. 1935;18(6):643.

[35] Wechsler D. Wechsler Adult Intelligence Scale: WAIS-IV; Technical and Interpretive Manual. Pearson; 2008.

[36] Kerchner GA, Racine CA, Hale S, et al. Cognitive processing speed in older adults: relationship with white matter integrity. PLoS One. 2012;7(11):e50425. 23185621 10.1371/journal.pone.0050425 PMC3503892

[37] Saloner R, Fonseca C, Paolillo EW, et al. Combined effects of synaptic and axonal integrity on longitudinal gray matter atrophy in cognitively unimpaired adults. Neurology. 2022;99(20):e2285-93. 36041868 10.1212/WNL.0000000000201165 PMC9694840

[38] Ashburner J, Friston KJ. Unified segmentation. Neuroimage. 2005;26(3):839–51. 15955494 10.1016/j.neuroimage.2005.02.018

[39] Desikan RS, Ségonne F, Fischl B, et al. An automated labeling system for subdividing the human cerebral cortex on MRI scans into gyral based regions of interest. NeuroImage. 2006;31(3):968–80. 16530430 10.1016/j.neuroimage.2006.01.021

[40] Schmidt P. Bayesian inference for structured additive regression models for large-scale problems with applications to medical imaging. Published online January 19, 2017.

[41] Orlhac F, Eertink JJ, Cottereau AS, et al. A Guide to ComBat Harmonization of Imaging Biomarkers in Multicenter Studies. J Nucl Med. 2022;63(2):172–9. 34531263 10.2967/jnumed.121.262464 PMC8805779

[42] McDonald GC. Ridge regression. Wiley Interdiscip Rev Comput Stat. 2009;1(1):93–100.

[43] Kuhn M. Building predictive models in R using the caret package. J Stat Softw. 2008;28(5):1–26.27774042

[44] Geidl W, Schlesinger S, Mino E, Miranda L, Pfeifer K. Dose-response relationship between physical activity and mortality in adults with noncommunicable diseases: a systematic review and meta-analysis of prospective observational studies. Int J Behav Nutr Phys Act. 2020;17(1):109. 10.1186/s12966-020-01007-5 PMC7448980 32843054

[45] Arem H, Moore SC, Patel A, et al. Leisure Time Physical Activity and Mortality: A Detailed Pooled Analysis of the Dose-Response Relationship. JAMA Intern Med. 2015;175(6):959. 25844730 10.1001/jamainternmed.2015.0533 PMC4451435

[46] Veldsman M, Tai XY, Nichols T, et al. Cerebrovascular risk factors impact frontoparietal network integrity and executive function in healthy ageing. Nat Commun. 2020;11(1):1–10. 32895386 10.1038/s41467-020-18201-5 PMC7477206

[47] Vemuri P, Graff-Radford J, Lesnick TG et al. White matter abnormalities are key components of cerebrovascular disease impacting cognitive decline. Brain Commun. 2021;3(2). 10.1093/braincomms/fcab076 PMC8072521 33937772

[48] Egginton S. Invited review: activity-induced angiogenesis. Pflugers Arch. 2009;457(5):963–77. 18704490 10.1007/s00424-008-0563-9

[49] Hambrecht R, Adams V, Erbs S, et al. Regular physical activity improves endothelial function in patients with coronary artery disease by increasing phosphorylation of endothelial nitric oxide synthase. Circulation. 2003;107(25):3152–8. 12810615 10.1161/01.CIR.0000074229.93804.5C

[50] Kleinloog JPD, Nijssen KMR, Mensink RP, Joris PJ. Effects of physical exercise training on cerebral blood flow measurements: a systematic review of human intervention studies. Int J Sport Nutr Exerc Metab. 2022;33(1):47–59. 36170974 10.1123/ijsnem.2022-0085

[51] Lopes S, Afreixo V, Teixeira M, et al. Exercise training reduces arterial stiffness in adults with hypertension: A systematic reviewand meta-analysis. J Hypertens. 2021;39(2):214–22. 32833924 10.1097/HJH.0000000000002619

[52] Nimmo MA, Leggate M, Viana JL, King JA. The effect of physical activity on mediators of inflammation. Diabetes Obes Metab. 2013;15(S3):51–60. 24003921 10.1111/dom.12156

[53] Byberg L, Zethelius B, McKeigue PM, Lithell HO. Changes in physical activity are associated with changes in metabolic cardiovascular risk factors. Diabetologia. 2001;44(12):2134–9. 11793014 10.1007/s001250100022

[54] Butt TH, Tobiume M, Re DB, Kariya S. Physical exercise counteracts aging-associated white matter demyelination causing cognitive decline. Aging Dis. 2024;15(5):2136. 38377028 10.14336/AD.2024.0216 PMC11346408

[55] Burma JS, Bailey DM, Johnson NE, et al. Physiological influences on neurovascular coupling: a systematic review of multimodal imaging approaches and recommendations for future study designs. Exp Physiol. 2025;110(1):23–41. 39392865 10.1113/EP092060 PMC11689421

[56] Jor’dan AJ, Manor B, Iloputaife I, et al. Diminished Locomotor Control Is Associated With Reduced Neurovascular Coupling in Older Adults. Journals Gerontology: Ser A. 2020;75(8):1516–22. 10.1093/gerona/glz006 PMC7357586 30629129

[57] Gonneaud J, Moreau I, Felisatti F, et al. Men and women show partly distinct effects of physical activity on brain integrity. Alzheimer’s Dementia: Diagnosis Assess Disease Monit. 2022;14(1):e12302. 10.1002/dad2.12302 PMC8959639 35382233

[58] Barha CK, Davis JC, Falck RS, Nagamatsu LS, Liu-Ambrose T. Sex differences in exercise efficacy to improve cognition: a systematic review and meta-analysis of randomized controlled trials in older humans. Front Neuroendocrinol. 2017;46:71–85. 28442274 10.1016/j.yfrne.2017.04.002

[59] Barha CK, for the Health A, Best BCS. Sex-Specific Relationship Between Long-Term Maintenance of Physical Activity and Cognition in the Health ABC Study: Potential Role of Hippocampal and Dorsolateral Prefrontal Cortex Volume. Journals Gerontology: Ser A. 2020;75(4):764–70. 10.1093/gerona/glz093 PMC7931854 30958523

[60] Varma VR, Chuang YF, Harris GC, Tan EJ, Carlson MC. Low-intensity daily walking activity is associated with hippocampal volume in older adults. Hippocampus. 2015;25(5):605–15. 25483019 10.1002/hipo.22397 PMC4425252

[61] Berchtold NC, Kesslak JP, Pike CJ, Adlard PA, Cotman CW. Estrogen and exercise interact to regulate brain-derived neurotrophic factor mRNA and protein expression in the hippocampus. Eur J Neurosci. 2001;14(12):1992–2002. 11860494 10.1046/j.0953-816x.2001.01825.x

[62] Zhao D, Guallar E, Ouyang P, et al. Endogenous sex hormones and incident cardiovascular disease in post-menopausal women. J Am Coll Cardiol. 2018;71(22):2555–66. 29852978 10.1016/j.jacc.2018.01.083 PMC5986086

[63] Crandall CJ, Barrett-Connor E. Endogenous sex steroid levels and cardiovascular disease in relation to the menopause: a systematic review. Endocrinol Metab Clin North Am. 2013;42(2):227–53. 23702399 10.1016/j.ecl.2013.02.003

[64] Chen FT, Etnier JL, Chan KH, Chiu PK, Hung TM, Chang YK. Effects of exercise training interventions on executive function in older adults: a systematic review and meta-analysis. Sports Med. 2020;50(8):1451–67. 32447717 10.1007/s40279-020-01292-x PMC7376513

[65] Aghjayan SL, Bournias T, Kang C, et al. Aerobic exercise improves episodic memory in late adulthood: a systematic review and meta-analysis. Commun Med. 2022;2(1):1–11. 35603310 10.1038/s43856-022-00079-7 PMC9053291

[66] Zhidong C, Wang X, Yin J, Song D, Chen Z. Effects of physical exercise on working memory in older adults: a systematic and meta-analytic review. Eur Rev Aging Phys Act. 2021;18(1):1–15. 34535084 10.1186/s11556-021-00272-y PMC8447686

[67] Iso-Markku P, Aaltonen S, Kujala UM, et al. Physical activity and cognitive decline among older adults: a systematic review and meta-analysis. JAMA Netw Open. 2024;7(2):e2354285. 38300618 10.1001/jamanetworkopen.2023.54285 PMC10835510

[68] Gallardo-Gómez D, del Pozo-Cruz J, Noetel M, Álvarez-Barbosa F, Alfonso-Rosa RM, del Pozo Cruz B. Optimal dose and type of exercise to improve cognitive function in older adults: a systematic review and bayesian model-based network meta-analysis of RCTs. Ageing Res Rev. 2022;76:101591. 35182742 10.1016/j.arr.2022.101591

