# Supplementary material for: The active ingredients: physical activity features linked to healthy brain aging
Source: Alzheimers Res Ther. 2026 Mar 5;18:88. doi: 10.1186/s13195-026-01998-6 (PMC13094126; doi:10.1186/s13195-026-01998-6)
Supplement: Supplementary file 1 — Supplementary Material 1 [file 13195_2026_1998_MOESM1_ESM.docx]

**Supplementary materials**

*Defining physical activity sessions at varying cadence thresholds*

To validate our chosen session cadence threshold of ≥40 steps/min, and to understand how engagement in physical activity sessions differed depending on the cadence threshold used, we iteratively tested different definitions of physical activity sessions using minimum cadence thresholds of 60, 80, 100 and 120 steps/min (i.e., at least 10 minutes where step cadence ≥60 steps/min, and so on). ANCOVAs compared cognitive and neuroimaging outcomes in participants who did and did not engage in at least one physical activity session defined by these different thresholds.

These analyses revealed that when the cadence threshold for defining a physical activity session was incrementally increased (i.e., from 40 to 60 steps/min and so on; Table S1), the association between physical activity sessions and lower WMH burden persisted regardless of threshold, though this relationship weakened at the highest cadence threshold of 120 steps/min. The relationship between engagement in sessions and global FA was not significant at lower cadence thresholds (40-80 steps/min), but showed a significant positive effect when sessions were defined with higher cadence thresholds of 100 and 120 steps/min. In addition, there was only a significant positive relationship between engagement in sessions and medial temporal lobe volume when sessions were defined at the highest cadence threshold of 120 steps/min. There was no statistically significant relationship between engagement in sessions at any cadence threshold for memory (all p >0.346), executive function (all p >0.133), processing speed (all p >0.447) or frontal lobe volume (all p >0.111).

**Table S1**Differential relationships between engagement in physical activity sessions with brain health outcomes at varying session cadence thresholds

|  | Physical activity session cadence threshold (steps/min) | | | | |
| --- | --- | --- | --- | --- | --- |
|  | Cadence = 40 | Cadence = 60 | Cadence = 80 | Cadence = 100 | Cadence = 120 |
| Memory (n = 197) | n(S) = 150  M(diff) = 0.076 ηp^2^ *=* 0.004  *p* = 0.793 | n(S) = 138  M(diff) = 0.080 ηp^2^ *=* 0.001 *p* = 0.806 | n(S) = 121  M(diff) = 0.161 ηp^2^ *=* 0.008 *p* = 0.346 | n(S) = 65  M(diff) = 0.163 ηp^2^ *=* < 0.001 *p* = 0.680 | n(S) = 20  M(diff) = 0.304 ηp^2^ *=* 0.008 *p* = 0.361 |
| Executive function  (n = 266) | n(S) = 212  M(diff) = 0.158 ηp^2^ *=* 0.004 *p* = 0.687 | n(S) = 200  M(diff) = 0.133 ηp^2^ *=* 0.004  p = 0.791 | n(S) = 176  M(diff) = 0.148 ηp^2^ *=* 0.008  *p* = 0.555 | n(S) = 113  M(diff) = 0.355 ηp^2^ *=* 0.025  p = 0.133 | n(S) = 39  M(diff) = 0.302 ηp^2^ *=* 0.012  p = 0.202 |
| Processing  speed (n = 203) | n(S) = 156  M(diff) = -0.019 ηp^2^ *=* < 0.001  *p* = 0.842 | n(S) = 147  M(diff) = 0.088 ηp^2^ *=* < 0.001  p = 0.917 | n(S) = 132  M(diff) = -0.191 ηp^2^ *=* 0.006  *p* = 0.447 | n(S) = 68  M(diff) = -0.137 ηp^2^ *=* 0.001  p = 0.759 | n(S) = 21  M(diff) = -0.077 ηp^2^ *=* < 0.001  p = 0.498 |
| MTL volume  (n = 182) | n(S) = 147  M(diff) = 607.64 ηp^2^ *=* 0.108 *p* = 0.228 | n(S) = 139  M(diff) = 517.25 ηp^2^ *=* 0.094  p = 0.384 | n(S) = 123  M(diff) = 309.42 ηp^2^ *=* 0.044  *p* = 0.351 | n(S) = 92  M(diff) = 222.80 ηp^2^ *=* 0.009  p = 0.703 | n(S) = 28  M(diff) = 348.34 ηp^2^ *=* 0.063  **p = 0.022** |
| Frontal lobe volume (n = 182) | n(S) = 147  M(diff) = 4432.44 ηp^2^ *=* 0.121 *p* = 0.121 | n(S) = 139  M(diff) = 3261.28 ηp^2^ *=* 0.080  p = 0.647 | n(S) = 123  M(diff) = 2844.48 ηp^2^ *=* 0.071  *p* = 0.111 | n(S) = 92  M(diff) = 1739.79 ηp^2^ *=* 0.014  p = 0.792 | n(S) = 28  M(diff) = -508.05 ηp^2^ *=* < 0.001  p = 0.848 |
| Total WMH burden* (n = 162) | n(S) = 130  M(diff) = -0.913 ηp^2^ *=* 0.126 ***p* = 0.005** | n(S) = 123  M(diff) = -0.757 ηp^2^ *=* 0.096  **p = 0.011** | n(S) = 110  M(diff) = -0.833 ηp^2^ *=* 0.138  **p = 0.001** | n(S) = 82  M(diff) = -0.817 ηp^2^ *=* 0.134  **p = 0.005** | n(S) = 26  M(diff) = -0.313 ηp^2^ *=* 0.045  p = 0.207 |
| Global FA  (n = 149) | n(S) = 116  M(diff) = 0.005 ηp^2^ *=* 0.009 *p* = 0.139 | n(S) = 108  M(diff) = 0.002 ηp^2^ *=* 0.002  p = 0.237 | n(S) = 96  M(diff) = 0.005 ηp^2^ *=* 0.014  p = 0.135 | n(S) = 66  M(diff) = 0.017 ηp^2^ *=* 0.099  **p = 0.005** | n(S) = 21  M(diff) = 0.017 ηp^2^ *=* 0.106  **p = 0.003** |

ANCOVA analyses controlled for age, sex, education and total intracranial volume where appropriate. n(S): number of participants who engaged in at least one session defined with the cadence threshold criteria; M(diff): mean group difference. Positive difference values indicate higher values in participants who engaged in sessions compared to those who did not. *Total WMH burden values were log transformed due to distribution skew. Statistically significant group differences (p<0.05) are shaded in grey.

**Table S2**Linear regression analyses between PA features and brain health outcomes

|  | **Medial temporal lobe volume** | | | **Frontal lobe volume** | | |
| --- | --- | --- | --- | --- | --- | --- |
| **Predictor** | **β (SE)** | ***p*** | ***p^FDR^*** | **β (SE)** | ***p*** | ***p^FDR^*** |
| Daily out-of-session steps | 0.12 (0.05) | **0.023** | 0.059 | 0.10 (0.05) | 0.063 | 0.219 |
| Daily in-session steps | 0.11 (0.06) | **0.035** | 0.059 | 0.02 (0.05) | 0.759 | 0.759 |
| Daily vigorous out-of-session steps | 0.07 (0.05) | 0.201 | 0.235 | 0.05 (0.05) | 0.350 | 0.490 |
| Daily vigorous in-session steps | 0.13 (0.05) | **0.008** | 0.059 | 0.07 (0.05) | 0.120 | 0.280 |
| Avg session duration | 0.05 (0.05) | 0.329 | 0.329 | 0.07 (0.05) | 0.215 | 0.377 |
| Avg session cadence | 0.12 (0.06) | **0.042** | 0.059 | 0.12 (0.06) | **0.047** | 0.219 |
| Avg session frequency | 0.13 (0.06) | **0.030** | 0.059 | 0.03 (0.06) | 0.605 | 0.706 |
|  | **Total WMH burden*** | | | **Global fractional anisotropy** | | |
| Daily out-of-session steps | -0.08 (0.06) | 0.177 | 0.206 | 0.06 (0.08) | 0.466 | 0.543 |
| Daily in-session steps | -0.12 (0.06) | **0.031** | 0.054 | 0.22 (0.08) | **0.009** | **0.030** |
| Daily vigorous out-of-session steps | -0.04 (0.05) | 0.416 | 0.416 | -0.04 (0.12) | 0.734 | 0.734 |
| Daily vigorous in-session steps | -0.09 (0.05) | 0.074 | 0.103 | 0.18 (0.07) | **0.013** | **0.030** |
| Avg session duration | -0.17 (0.06) | **0.006** | **0.040** | 0.22 (0.08) | **0.006** | **0.030** |
| Avg session cadence | -0.15 (0.06) | **0.019** | 0.054 | 0.20 (0.08) | **0.019** | **0.034** |
| Avg session frequency | -0.14 (0.06) | **0.029** | 0.054 | 0.16 (0.08) | 0.065 | 0.091 |
|  | **Memory** | | | **Executive function** | | |
| Daily out-of-session steps | 0.13 (0.07) | 0.083 | 0.193 | 0.01 (0.06) | 0.818 | 0.818 |
| Daily in-session steps | 0.09 (0.08) | 0.279 | 0.488 | 0.21 (0.06) | **<0.001** | **0.001** |
| Daily vigorous out-of-session steps | 0.27 (0.11) | **0.015** | 0.108 | 0.12 (0.06) | **0.039** | 0.054 |
| Daily vigorous in-session steps | 0.18 (0.08) | **0.036** | 0.126 | 0.20 (0.05) | **<0.001** | **0.001** |
| Avg session duration | 0.05 (0.07) | 0.501 | 0.585 | 0.08 (0.06) | 0.196 | 0.229 |
| Avg session cadence | 0.05 (0.08) | 0.497 | 0.585 | 0.18 (0.06) | **0.006** | **0.010** |
| Avg session frequency | 0.02 (0.08) | 0.809 | 0.809 | 0.18 (0.06) | **0.003** | **0.007** |
|  | **Spatial info processing speed** | | |  |  |  |
| Daily out-of-session steps | -0.04 (0.07) | 0.624 | 0.728 |  |  |  |
| Daily in-session steps | -0.09 (0.08) | 0.235 | 0.412 |  |  |  |
| Daily vigorous out-of-session steps | -0.20 (0.10) | **0.047** | 0.155 |  |  |  |
| Daily vigorous in-session steps | -0.13 (0.07) | 0.056 | 0.155 |  |  |  |
| Avg session duration | -0.02 (0.07) | 0.785 | 0.785 |  |  |  |
| Avg session cadence | -0.15 (0.08) | 0.066 | 0.155 |  |  |  |
| Avg session frequency | -0.06 (0.07) | 0.391 | 0.547 |  |  |  |

Each model statistic was obtained from the result of a separate linear regression analysis examining the relationship between a single predictor and the outcome. Models covaried for age and sex (all outcomes), education (cognitive outcomes) and total intracranial volume (neuroimaging outcomes). p values are presented with and without false discovery rate (FDR) correction. Bolded p values represent statistical significance of p < 0.05. *Total WMH burden values were log transformed due to distribution skew.

**Table S3**

Coefficient rankings from ridge regression models excluding interaction terms

| **WMH burden** | **Global FA** | **MTL volume** |
| --- | --- | --- |
| Model R^2^ = 0.042, MAE = 0.688  Feature ranking:   1. Session frequency 2. Session cadence 3. Session duration 4. In-session vig steps 5. Out-of-session vig steps 6. Total in-session steps 7. Total out-of-session steps | Model R^2^ = 0.107, MAE = 0.652  Feature ranking:   1. Session cadence 2. Session duration 3. Session frequency 4. Out-of-session vig steps 5. In-session vig steps 6. Total in-session steps 7. Total out-of-session steps | Model R^2^ = 0.050, MAE = 0.766  Feature ranking:   1. Session frequency 2. Session cadence 3. Session duration 4. Out-of-session vig steps 5. In-session vig steps 6. Total in-session steps 7. Total out-of-session steps |
| **Frontal volume** | **Executive Function** | **Memory** |
| Model R^2^ = 0.038, MAE = 0.799  Feature ranking:   1. Session cadence 2. Session duration 3. Session frequency 4. Out-of-session vig steps 5. In-session vig steps 6. Total out-of-session steps 7. Total in-session steps | Model R^2^ = 0.077, MAE = 0.519  Feature ranking:   1. Session frequency 2. Session cadence 3. Session duration 4. Out-of-session vig steps 5. In-session vig steps 6. Total in-session steps 7. Total out-of-session steps | Model R^2^ = 0.056, MAE = 0.609  Feature ranking:   1. Session frequency 2. Session duration 3. Out-of-session vig steps 4. Session cadence 5. In-session vig steps 6. Total out-of-session steps 7. Total in-session steps |
| **Processing speed** |  |  |
| Model R^2^ = 0.029, MAE = 0.773  Feature ranking:   1. Session frequency 2. Session cadence 3. Session duration 4. Out-of-session vig steps 5. In-session vig steps 6. Total in-session steps 7. Total out-of-session steps |  |  |

In sensitivity analyses, each ridge regression model was run with only the seven core physical activity features excluding interaction terms with age and sex. Vig: vigorous


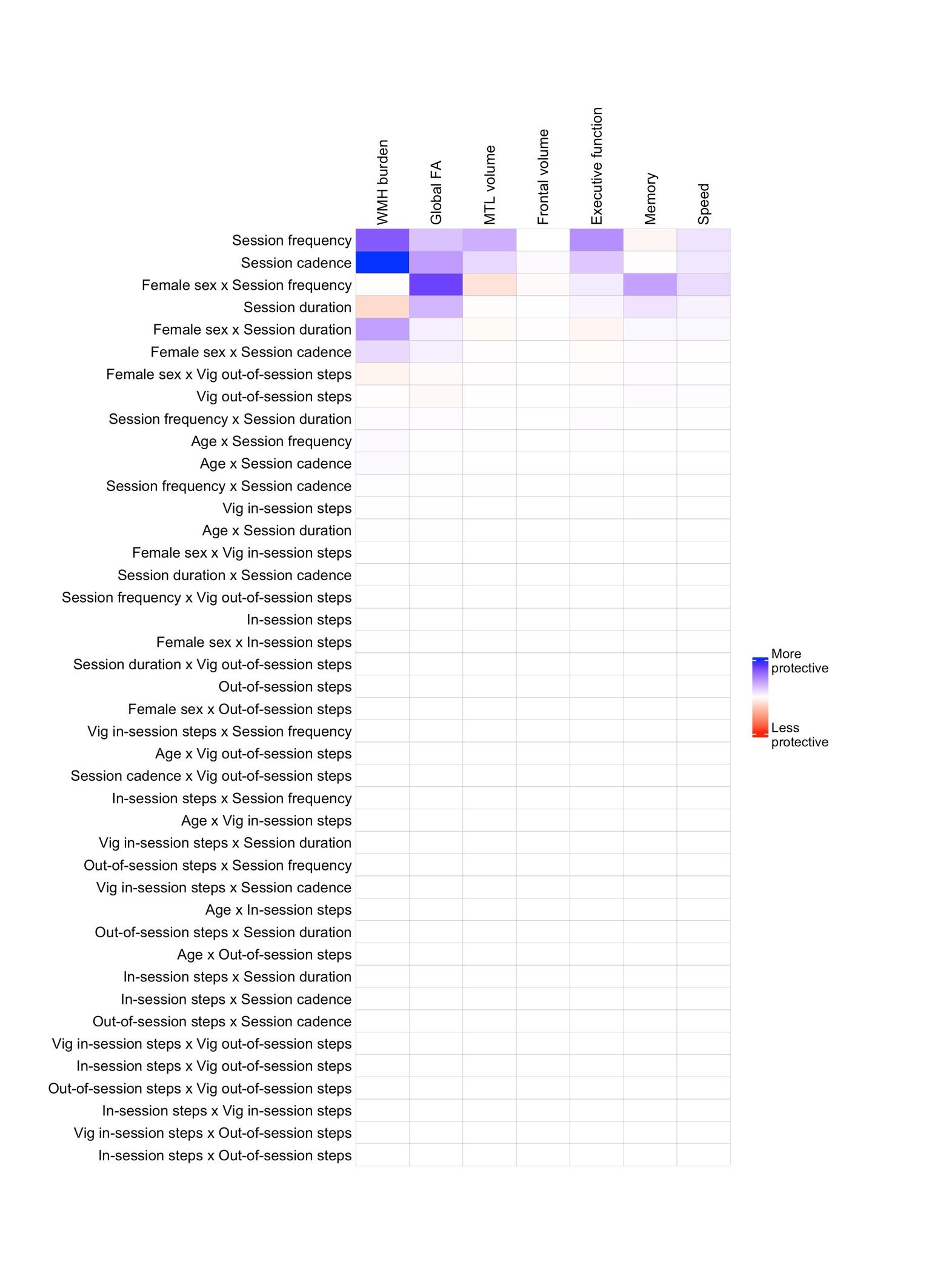


**Figure S1.** A full heat map of standardized coefficients from ridge regression models examining the importance of PA features on brain health outcomes in exercisers. Numerical standardized coefficient values are uninterpretable in ridge regression models due to regularization and are therefore not presented. Instead, the strength of standardized coefficients is interpretable relative to other coefficients in the model (i.e. rank order). Rows of the heatmap are ordered to reflect the average importance ranking of PA session features across outcomes in descending order (top to bottom). Outcomes were pre-adjusted for demographic covariates including age, sex, education and total intracranial volume. WMH: white matter hyperintensity; FA: fractional anisotropy; Vig: vigorous.
